# Bariatric surgery: Potential post-operative heightened sensitivity to substances or behaviors

**Published:** 2021-03-19

**Authors:** Panayotis K. Thanos, Mark S. Gold, Kenneth Blum, Rajendra D Badgaiyan, Nicole M. Avena

**Affiliations:** 1 University at Buffalo, Department of Pharmacology and Toxicology, Buffalo, NY, USA; 2 Washington University in St Louis School of Medicine, St. Louis, MO, USA; 3 Graduate College, Western University Health Sciences, Pomona, CA, USA; 4 Departments of Psychiatry and Neuroscience, Icahn School of Medicine at Mount Sinai. NYC, NY, USA

It is well-known that approximately 20% of patients who underwent Roux-en-Y gastric bypass (RYGB) and 11% of patients who undertook laparoscopic adjustable gastric banding acquired AUD within five years of surgery. Notably, decreases in BMI following RYGB have decreased food addiction symptoms (as measured by the Yale Food Addiction Scale) and increased alcohol intake 24 months post-operation. After many years of successful bariatric (weight-loss) surgeries targeted at the obesity epidemic, clinicians report that some patients replace compulsive overeating with newly acquired compulsive disorders such as alcoholism, gambling, drugs, and other addictions like compulsive shopping and exercise. Our laboratory previously coined the term Reward Deficiency Syndrome (RDS) for common genetic determinants in predicting addictive disorders and reported that the Bayesian predictive value for future RDS behaviors in subjects carrying the DRD2 Taq A1 allele was 74% [1]. Transfer potential, data from our laboratory has shown that in an obese cohort undergoing bariatric surgery based on genetic addiction risk testing showed that these individuals carried a high risk for illicit drug abuse as well as Alcohol Use Disorder (AUD) suggestive of addiction transfer potential.

Arterburn, *et al.* [2] summarized the literature on bariatric surgery benefits and risks. Obesity and associated health consequences remain the largest public health epidemic causing preventable death. Arterburn et al. examined the current epidemiology of obesity and bariatric surgical procedures and the beneficial effects on numerous obesity-related comorbidities (e.g., type 2 diabetes, dyslipidemia, hypertension, sleep apnea, and cancer), in addition to long-term weight loss.

However, an important point not widely discussed was that with improved food choices and reduced food cravings following bariatric surgery, some patients developing a greater vulnerability to substance abuse or an increased risk for alcohol consumption and alcohol use disorder (AUD) [3]. Specifically, 20% of patients who underwent Roux-en-Y gastric bypass (RYGB) and 11% of patients who underwent laparoscopic adjustable gastric banding showed AUD within five years of surgery [4]. Decreases in BMI following RYGB have associated with decreased food addiction symptoms (as measured by the Yale Food Addiction Scale) but increased alcohol intake 24 months post-op [5].

Similarly, preclinical data, not confounded by psychosocial and comorbidity factors specific to humans, have also shown increased alcohol preference and intake in dietary obese rats following RYGB [6], but not observed in rodents following vertical sleeve gastrectomy. Hormonal changes (ghrelin) may play a role in the differential AUD risks between bariatric surgical procedures. Preclinical evidence supports that ghrelin is involved in alcohol-seeking as it modulates both central reward and stress pathways. Neuroimaging data revealed that ghrelin modulated the food-related signal in the medial orbitofrontal cortex and nucleus accumbens and increased the alcohol-related signal in the amygdala [7].

According to the ‘symptom substitution’ theory, “the successful elimination of a particular symptom without treating the underlying cause will result in the appearance of a substitute symptom” [8]. Therefore, following bariatric surgery, an increase in alcohol or substance use, in general, might follow because the surgery largely eliminates hyperphagia without altering individual vulnerabilities to addictive disorders. Also, following bariatric surgery, food cues may elicit reduced activation in brain reward areas and a blunted activation in the prefrontal cortex, an area involved in inhibiting impulsive behaviors. In-part these food cues and subsequent blunted response may be due to specific genetic antecedents, including the DRD2 A1 allele, as reported in a series of human studies by Stice, *et al.* [9]. It has been theorized with some clinical data that individuals with hypofunctioning reward circuitry overeat to compensate for a reward deficit, obese compared to lean humans have a small number of striatal D2 receptors and show less striatal response to palatable food intake.

As stated earlier, after many years of successful bariatric (weight-loss) surgeries targeted at the obesity epidemic, clinicians report that some patients replace compulsive overeating with newly acquired compulsive disorders such as alcoholism, gambling, drugs, and other addictions like compulsive shopping and exercise [10]. Previously Blum et al. coined the term Reward Deficiency Syndrome (RDS) for common genetic determinants in predicting addictive disorders and reported that the Bayesian predictive value for future RDS behaviors in subjects carrying the DRD2 Taq A1 allele was 74% [11]. Previously, the hypothesis that RDS is the root cause of substituting food addiction for other dependencies and potentially explains this recently described Phenomenon (addiction transfer) common after bariatric surgery [1]. Importantly data from our laboratory has shown that an obese cohort undergoing bariatric surgery based on genetic addiction risk testing showed that these individuals carried a high risk for illicit drug abuse as well as Alcohol Use Disorder (AUD) suggestive of addiction transfer potential [1].

Given the similarities in vulnerabilities and mechanisms between obesity and drug addiction collectively associated with addiction and reward deficiency syndrome [3], future use of neuroimaging, genetic and psychosocial testing could shed light on these hypotheses and advance precision behavioral medicine approach. Improved patient outcomes following bariatric surgery would include behavioral interventions and counseling on the potential post-operative heightened sensitivity to substances or behaviors as a food replacement or coping mechanism.

## References

[1] Blum K, Wood RC, Braverman ER, Chen TJ, Sheridan PJ, (1995) The D2 dopamine receptor gene as a predictor of compulsive disease: Bayes’ theorem. Funct Neurol 10: 37–44. 7649500

[2] Arterburn DE, Telem DA, Kushner RF, Courcoulas AP (2020) Benefits and Risks of Bariatric Surgery in Adults: A Review. JAMA 324: 879–887. 32870301 10.1001/jama.2020.12567

[3] Blum K, Thanos PK, Gold MS (2019) Effects of hunger on mood and affect reactivity to monetary reward in women with obesity – A pilot study. Front Psychol 5: 919. 10.1371/journal.pone.0232813 PMC7237012 32428002

[4] Blum K, Wood RC, Braverman EP, Chen TJH, Sheridan PJ, (1995) D2 dopamine receptor gene as a predictor of compulsive disease: Bayes’ theorem. Functional Neurology 10: 37–44. 7649500

[5] Farokhnia M, Grodin EN, Lee MR, Oot EN, Blackburn AN, (2018) Exogenous ghrelin administration increases alcohol self-administration and modulates brain functional activity in heavy-drinking alcohol-dependent individuals. Mol Psychiatry 23: 2029–2038. 29133954 10.1038/mp.2017.226 PMC12258500

[6] Kazdin AE (1982) Symptom substitution, generalization, and response covariation: Implications for psychotherapy outcome. Psychol Bull 91: 349–365. 7071264

[7] King WC, Chen JY, Courcoulas AP, Dakin GF, Engel SG, (2017) Alcohol and other substance use after bariatric surgery: prospective evidence from a U.S. multicenter cohort study. Surg Obes Relat Dis 13: 1392–1402. 28528115 10.1016/j.soard.2017.03.021 PMC5568472

[8] Murray SM, Twerdy S, Geliebter A, Avena NM (2019) A Longitudinal Preliminary Study of Addiction-Like Responses to Food and Alcohol Consumption Among Individuals Undergoing Weight Loss Surgery. Obes Surg 29: 2700–2703. 31147822 10.1007/s11695-019-03915-3

[9] Stice E, Yokum S, Blum K, Bohon C (2010) Weight Gain Is Associated with Reduced Striatal Response to Palatable Food. J Neurosci 30: 13105–13109. 20881128 10.1523/JNEUROSCI.2105-10.2010 PMC2967483

[10] Thanos PK, Subrize M, Delis F, Cooney RN, Culnan D, (2012) Gastric bypass increases ethanol and water consumption in diet-induced obese rats. Obes Surg 22: 1884–1892. 22976430 10.1007/s11695-012-0749-2 PMC3615544

[11] Orellana E, Jamis C, Horvath N, Hajnal A (2018) Effect of vertical sleeve gastrectomy on alcohol consumption and preferences in dietary obese rats and mice: A plausible role for altered ghrelin signaling. Brain Res Bull 138: 26–36. 28802901 10.1016/j.brainresbull.2017.08.004 PMC6537102

